# First cavernicolous trechine beetle discovered in Guilin karst, northeastern Guangxi (Coleoptera, Carabidae, Trechinae)

**DOI:** 10.3897/zookeys.545.6111

**Published:** 2015-12-14

**Authors:** Feifei Sun, Mingyi Tian

**Affiliations:** 1 Department of Entomology, College of Agriculture, South China Agricultural University, no. 483 Wushan Road, Guangzhou, Guangdong, 510642, China; 2 Agricultural Bureau, Fengshun County, Guangdong, 514300, China

**Keywords:** Ground beetle, subterranean, new subgenus, new species, China

## Abstract

A new subgenus and new species of anophthalmic trechine beetles, Oodinotrechus (Pingleotrechus) yinae **subgen. n., sp. n.**, is described and illustrated from a limestone cave called Chaotianyan in southern part of Guilin karst, northeastern Guangxi Zhuang Autonomous Region. The new taxon is very different from the Maolan-Mulun congeners belonging to the nominate subgenus *Oodinotrechus* (*s. str.*) Uéno, 1998, in several important character states including pronotal structure, elytral chaetotaxy and male genitalia. It is the first record of a cavernicolous trechine beetle in Guilin karst, and in the eastern part of Guangxi. In addition, a distribution map for the genus *Oodinotrechus* Uéno, 1998, is provided.

## Introduction

Many karstic landscapes and limestone caves are distributed in southern China, the largest karstic area in the world (Waltham 2009; Lu 2012). Guilin is one of the most famous karstic wonders in China and is listed in the World Heritage as part of South China Karst (UNESCO 2015). Guilin karst ranges from 110°09' to 110°42'E and 24°40' to 25°40'N, with total area of 7104 km^2^ in which 3752 km^2 ^are carbonate rocks and represented mainly by Fenglin landscapes (Zhu 1988).

However, the cave fauna of the Guilin karst is not well-known. Wang and Cao (1998) reported the fauna in several caves in Guilin area, which is rather poor comparing to Mulun karst (Deharveng et al. 2008). Regarding Coleoptera, only one beetle species belonging to the family Pselaphidae was recorded in Guilin karst (Nomura and Wang 1991).

The anophthalmic cavernicolous trechine genus *Oodinotrechus* Uéno, 1998, was erected to contain the single species, *Oodinotrechus kishimotoi* Uéno, 1998, found in a cave in Maolan karst of southernmost Guizhou Province. So far, it is known by only the holotype, a male exemplar (Uéno 1998). Then, a second species, *Oodinotrechus liyoubangi* Tian, 2014, was discovered in several caves in Mulun karst, Huanjiang County, northernmost Guangxi (Tian 2014). Mulun karst is connected to Maolan karst to the south, and both karsts share the most beautiful karstic forest in southern China and have similar cave fauna.

**Figure 1.**
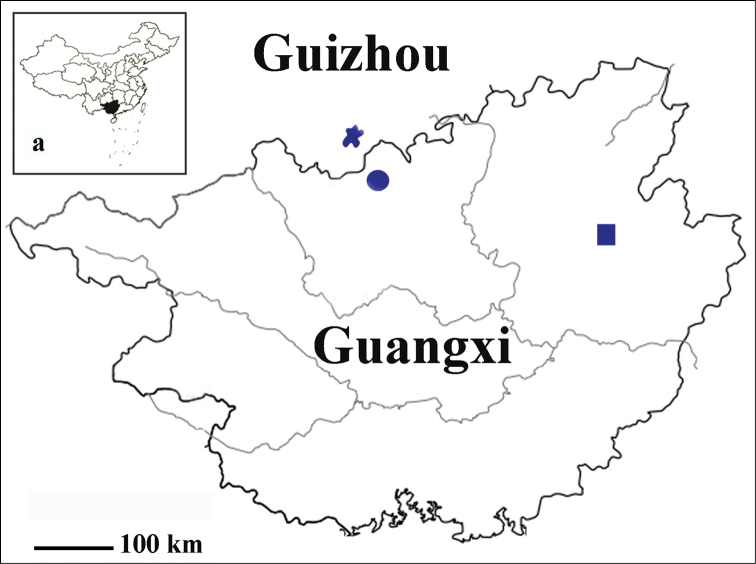
Distribution of the genus *Oodinotrechus* Uéno, 1998 ★ Oodinotrechus (Oodinotrechus) kishimotoi Uéno ● Oodinotrechus (Oodinotrechus) liyoubangi Tian, 2014 ■ Oodinotrechus (Pingleotrechus) yinae subgen. n., sp. n. **a** map of China, showing the location of Guangxi Zhuang Autonomous Region.

During two visits in the limestone cave called Chaotianyan, Pingle County, southern part of Guilin karst, a number of exemplars of a quite small sized beetle belonging to *Oodinotrechus* were collected. Further study confirmed that it is a new species and the first troglobitic trechine found in Guilin karst. Because of its striking characteristics which are so different from other congeners occurring Maolan-Mulun karst, a new subgenus named *Pingleotrechus* subgen. n. is established to accommodate this new species. To date, *Oodinotrechus* contains three species and ranges from southernmost Guizhou / northernmost Guangxi to northeastern Guangxi (Fig. 1).

## Materials and methods

The beetles for this study were collected by hand or by using an aspirator, and kept in 55% ethanol before study. Dissections and observations were made under a Leica MZ75 dissecting microscope. Dissected genital pieces, including the median lobe and parameres of aedeagus, were glued on small paper cards and then pinned under the specimen from which they were removed. Digital pictures were taken using a Canon EOS 40D camera, and then processed by means of Adobe Photoshop CS5 software.

Length of body is measured from apex of right mandible (in opened position) to elytral apex.

**Abbreviations of other measurements used in the text are as following**:

HLm length of head including mandibles, from apex of right mandible to occipital suture

HLl length of head excluding mandibles, from front of labrum to occipital suture

HW maximum width of head

PL length of pronotum, along the median line

PW maximum width of pronotum

PfW width of pronotum at front

PbW width of pronotum at base, measured between hind angles

EL length of elytra, from base of scutellum to elytral apex

EW maximum width of combined elytra

**Abbreviations for the specimens’ depository are as following**:

IOZ National Museum of Zoology, Institute of Zoology, Chinese Academy of Sciences, Beijing

MNHN Muséum National d’Histoire Naturelle, Paris

SCAU South China Agricultural University, Guangzhou

ZUBM Biological Museum of Zhongshan University, Guangzhou

## Taxonomic treatments

### Pingleotrechus subgen. n.

Taxon classificationAnimaliaColeopteraCarabidae

http://zoobank.org/B33BD019-3F0F-4CA7-8767-D0FA758DEC91

#### Type species.

*Oodinotrechus yinae* sp. n. (Cave Chaotianyan, Pingle County)

#### Diagnosis.

Similar to the nominate subgenus *Oodinotrechus* (*s. str.*) Uéno, 1998, but smaller and slenderer; body, in particular head and elytra, longer; head narrower, genae only slightly expanded laterally; pronotal base with sides much in advance of nearly straight medial part, without a gap submedially on each side between pronotum and elytra; scutellum visible from above; elytra slenderer, with two dorsal pores on 3^rd^ and 4^th^ striae, respectively; umbilical setae 5 and 6 widely separate, distance between them almost triple as that between setae 4 and 5; an additional striole running inside and forming a crescent with apical stria; aedeagus short and stout, without sagittal aileron.

#### Remarks.

*Pingleotrechus* subgen. n. is similar to *Oodinotrechus* (*s. str.*) Uéno, 1998, occurring in Maolan-Mulun karst, in many aspects, *viz.* short and stout body, short legs and antennae, entire frontal furrows, tridentate right mandible, well-defined labial suture, two pairs of supraorbital setigerous pores present on head, campanulate pronotum, ciliate elytral margin, presence of two dorsal pores on elytron, and unmodified protarsi in male. However, *Pingleotrechus* is different from the nominate subgenus in many characters as follows: body smaller and slenderer; head slightly expanded laterally (versus strongly expanded); second dorsal pore situated on 4^th^ elytral stria (versus on 5^th^ stria); umbilicate setigerous pores of the middle group (pores 5 and 6) widely separated (versus very close to each other); an additional, inner, apical striole, present (versus absent); male genitalia stouter, without sagittal aileron (versus with a large sagittal aileron).

#### Etymology.

Refers to Pingle County, the locality of the type species.

#### Distribution.

China (northeastern Guangxi) (Fig. 1).

### Oodinotrechus (Pingleotrechus) yinae sp. n.

Taxon classificationAnimaliaColeopteraCarabidae

http://zoobank.org/0094B29C-B668-495F-9325-952D4E85B0F2

**Figure 2.**
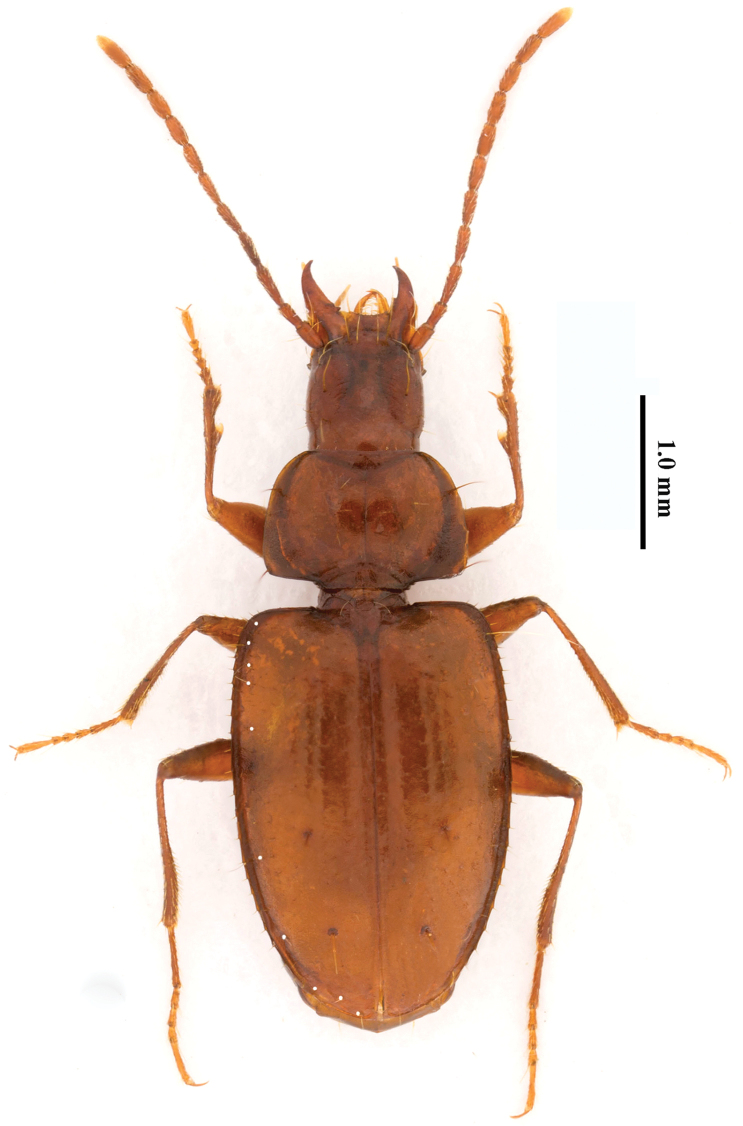
Oodinotrechus (Pingleotrechus) yinae subgen. n., sp. n. Habitus, holotype, male (chaetotaxy indicated by small white round points on left part of the body) (photos Xinhui Wang).

**Figures 3–4.**
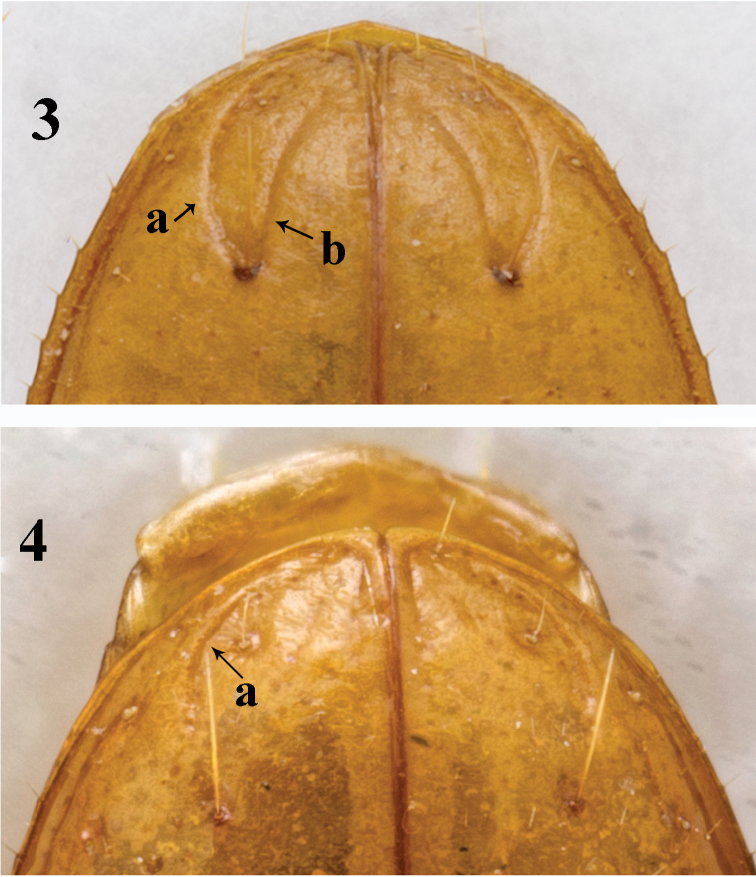
*Oodinotrechus* species, apical part of elytra **3** Oodinotrechus (Pingleotrechus) yinae subgen. n., sp. n. **4** Oodinotrechus (Oodinotrechus) liyoubangi **a** apical stria **b** additional striola.

**Figures 5–6.**
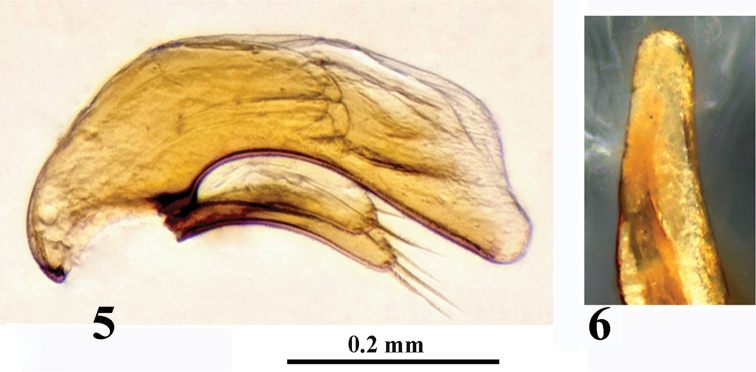
Oodinotrechus (Pingleotrechus) yinae subgen. n., sp. n., aedeagus **5** median lobe and parameres, lateral view **6** apical lobe of median lobe, dorsal view (photos Xinhui Wang).

Figs 1 , 2 , 3 , 5–6

#### Diagnosis.

A small, short and stout beetle, anophthalmic and depigmented, with short appendages, fore body distinctly shorter than elytra, head short and narrow, with two pairs of supraorbital setae, mentum and submentum separated by clear labial suture, pronotum broad and campanulate, both lateromarginal setae present, elytra serrate and ciliate, shoulders distinct, 5^th^ and 6^th ^pores of the marginal umblicate series widely separated, a crescent-form structure present on apical part of each elytron.

#### Description.

Length: 4.0–4.8 mm; width: 1.3–1.7 mm. Habitus as in Fig. 2.

Depigmented; yellowish to reddish dark brown, very shiny, palps and tarsi light yellow.

Genae with several sparse and short setae, frons and vertex glabrous; a pair of suborbital setae present between mentum and prosternum; disc of pronotum with four erect setae each side of median line; propleura and mesosternum glabrous, pro- and metasterna with a few setae medially; elytra, prosternum and abdominal ventrites sparsely pubescent, entire legs pubescent. Microsculpture formed by faintly engraved transverse meshes on head and elytra, moderately transverse on pronotum.

Head anophthalmic, subquadrate, distinctly longer than wide, HLm/HW=1.74–1.79, HLl/HW=1.22–1.29; frons depressed and almost flat, vertex slightly convex, frontal furrows long and deep. Genae slightly expanded laterally, distance between anterior and posterior supraorbital pores almost as great as minimum distance between frontal furrows. Mentum bisetose, with medial tooth simple and blunt. Submentum with a row of 6 setae. Palps slender, 3^rd^ maxillary palpomere as long as 4^th^ while 2^nd^ labial palpomere longer than 3^rd^. Antennae short and subfiliform, extended to about basal 1/3 of elytra and pubescent from 2^nd^ antennomere; 1^st^ antennomere with several additional setae near apex, slightly longer than 2^nd^, 3^rd^ longest; antennomeres 4–10 decreasing gradually in length, 11^th^ as long as 4^th^.

Pronotum transversely campanulate, PW/PL=1.40–1.47, as long as head, widest just before hind angles, which are widely obtuse and not denticulate; sides gently and gradually converging apicad in a smooth arc; fore angles are rounded off; anterior margin nearly straight. Base nearly straight, markedly wider than apex, PWb/PWf=1.67–1.71, with sides in advance of median part and oblique towards hind angles; lateral margins widely expanded and reflexed; anterolateral seta a third from apex, posterolateral seta just in front of hind angle. Disc moderately convex. Median line fine and deep. Both frontal and basal transverse impressions well-marked, basal foveae indistinct.

Scutellum large, visible from above. Elytra longer than forebody, EL/(HLm+PL)=1.32–1.39, longer than wide, EL/EW=1.54–1.79, with unbordered base, rounded shoulders and narrowed apex; widest about 1/3 from base; lateral margin serrate and ciliate throughout. Disc moderately convex, distinctly depressed at base and along suture. Parascutellar striole absent. 1^st^ stria distinct, 2^nd^ and 3^rd^ faint but traceable; apical striole long and well-marked. Intervals flat, 2^nd^ wider than any other. Basal setigerous pore present, anterior dorsal pore on 3^rd^ stria about 1/6 from base, second pore on 4^th^ stria a little behind middle; preapical pore distinct, inserted at anterior end of apical striole about 1/4 from apex, where 3^rd^ and 4^th^ striae anastomosed. Umbilicate setigerous pores subdivided into subhumeral group (setae 1–4, closely spaced), middle group (setae 5 and 6, widely separated) and preapical group; seta 5 much closer to 4^th^ than to 6^th^, 6^th^ closer to 7^th^ than to 5^th^; distance between 5^th^ and 6^th^ about three times greater than between 5^th^ and 4^th^, preapical pore equidistant from suture and from apex of elytra. Apical stria (Fig. 3a) and an additional, inner, striole (Fig. 3b) rising from preapical pore and forming a crescent combined; this additional striole gently curved inwards behind and not quite reaching stria 1.

Protibia straight, without longitudinal external sulcus. Protarsomeres 2–4 nearly moniliform. 1^st^ tarsomere slightly shorter than or as long as, or longer than 2–4^th^ combined in protarsi, mesotarsi, and metatarsi, respectively.

Male genitalia (Figs 5–6): Aedeagus moderately sclerotized; median lobe small, short and stout, moderately arcuated in lateral view; ventral margin deeply concave in median portion, then gently and almost straight towards apex, which is rather broad, not tube-like. Basal portion very large, with a large basal orifice, sagittal aileron absent. Dorsal orifice wide and long. Inner sac armed with an indistinct copulatory piece, about 1/3 as long as aedeagus. Apical lamella in dorsal view rather long and thin, not parallel-sided, gently narrowed towards apex which is rounded at tip. Parameres short and narrow, right slightly longer than left, both are broadly rounded at apex and bearing three and four long setae, respectively.

#### Etymology.

This new species is named in honor of Ms. Haomin Yin, an active member in our cave biological study team.

#### Distribution.

**Figures 7–11.**
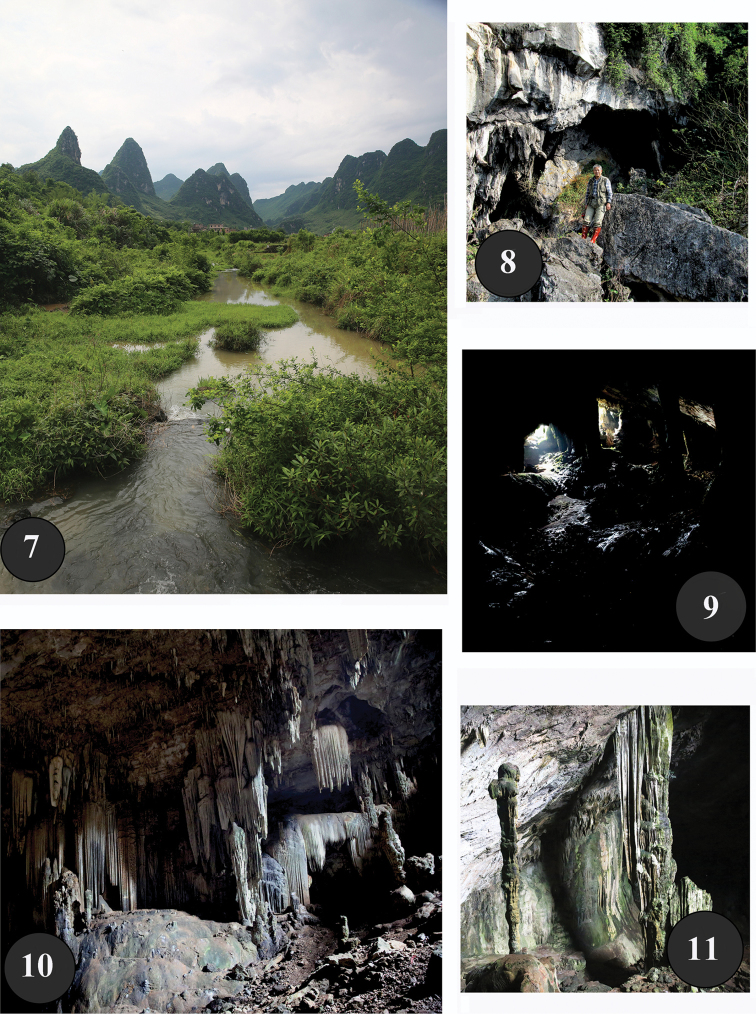
The type locality of the cave Chaotianyan and its surroundings **7** Fongchong landscape in Letang area **8** cave opening **9–11** a hall and deposits inside the cave (photos Mingyi Tian).

Northeastern Guangxi (Fig. 1). Known only from cave Chaotianyan, the type locality. The cave Chaotianyan is located in Letang village (Fig. 7), Ertang, Pingle County, at the southern part of Guilin karst. It opens in a subway of a hill, at about 60 m above foot, with a big entrance (Fig. 8). There is a large hall after the entrance, decorated by different deposits (Figs 9–11). It is a long cave according to the villagers, but detailed information is still not available. The beetles were collected in a dark and wet area under stone, not far from the entrance.

It is an interesting discovery to find an *Oodinotrechus* species in Guilin karst, northeastern Guangxi, because cave Chaotianyan, the type locality of *Oodinotrechus yinae* sp. n. is approximately 300 km away from the Maolan-Mulun karsts in the bordering areas between southernmost Guizhou and northernmost Guangxi where other two species of *Oodinotrechus* are found. However, in the Letang area there is a Fengchong landscape (Fig. 7), rather than Fenglin landscape which is dominant in other parts of the Guilin karst (Zhu 1988).

#### Materials examined.

Holotype: male, Guangxi: Guilin: Pingle: Ertang: Letang: cave Chaotianyan, 110°45’501"E / 24°37’075"N, 5-XII-2011, Mingyi Tian, Weixin Liu & Haomin Yin leg, in SCAU. Paratypes: 3 males and 4 females, same data as holotype; 6 males and 4 females, same cave, 29-IV-2013, Mingyi Tian, Weixin Liu, Haomin Yin & Feifei Sun leg. All are deposited in SCAU, except one male paratype in each of IOZ, MNHN and ZUBM, respectively.

### Key to species of the genus *Oodinotrechus* Uéno

**Table:** 

1	Body smaller and slenderer, head slightly expanded laterally; second dorsal pore situated on 4^th^ elytral stria, umbilical setae 5 and 6 widely separated; elytra in apical quarter with a crescent formed by apical striole proper and an additional, inner, striole; aedeagus stouter, without sagittal aileron (*Pingleotrechus* subgen. n.)	**Oodinotrechus (Pingleotrechus) yinae sp. n.**
–	Body mostly larger and stouter, head distinctly expanded laterally; second dorsal pore situated on 5^th^ elytral interval; umbilical setae 5 and 6 set close to each other; only apical striole present; aedeagus slenderer, with a large sagittal aileron (*Oodinotrechus* (s. str.))	**2**
2	Scutellum at least partly visible from above; basal margin near hind angle nearly straight; apex of the median lobe of aedeagus parallel-sided in dorsal view; both parameres bearing four long setae at apices	**Oodinotrechus (Oodinotrechus) liyoubangi Tian**
–	Scutellum invisible from above; basal margin near hind angle emarginate, apex of the median lobe of aedeagus slightly expanded and gradually narrowed in dorsal view, parameres each bearing two or three long apical setae	**Oodinotrechus (Oodinotrechus) kishomotoi Uéno**

## Supplementary Material

XML Treatment for Pingleotrechus

XML Treatment for Oodinotrechus (Pingleotrechus) yinae
